# Social networks are shaped by culturally contingent assessments of social competence

**DOI:** 10.1038/s41598-023-34723-6

**Published:** 2023-05-17

**Authors:** Sareena Chadha, Adam M. Kleinbaum, Adrienne Wood

**Affiliations:** 1 Department of Psychology, University of Virginia, 485 McCormick Rd, Charlottesville, VA 22904 USA; 2 Tuck School of Business, Dartmouth College, 100 Tuck Hall, Hanover, NH 03755 USA

**Keywords:** Psychology, Human behaviour

## Abstract

Cultural outsiders, like immigrants or international students, often struggle to make friends. We propose that one barrier to social connection is not knowing what it means to be socially competent in the host culture. First-year students at a U.S. business school (N = 1328) completed a social network survey and rated their own social competence and that of several peers. International students were rated by peers as less socially competent than U.S. students, especially if they were from nations more culturally dissimilar to the U.S. International students’ self-reported competence ratings were uncorrelated with peers’ judgments. Social network analysis revealed international students were less central to their peer networks than U.S. students, although this gap was reduced if peers evaluated them as socially competent. Peer-reported competence mediated the effects of international student status on social network centrality. Since learning local norms takes time, we suggest inclusivity will require host communities to define social competence more broadly.

## Introduction

Migration is an increasingly common part of life in the globalized age. An estimated 280 million people live in a nation other than their birthplace, an increase of 61% since 2000^1^. Fourteen percent of the U.S. population was born elsewhere^2^. Cultural outsider status in a new country presents several difficulties, such as navigating unfamiliar institutions, expectations, and social environments without an established social support network^3^. Finding local social support is essential for cultural outsiders’ long-term success^4^. Intercultural friendship improves international students’ academic performance, cultural adjustment, and social integration and support^5,6^.

Yet many cultural outsiders struggle to form relationships in their new environments. Typically, cultural outsiders feel lonelier and less socially satisfied than host nationals^3^. Cultural outsiders, whether in school or not, encounter numerous challenges to forming relationships with locals, including host nationals’ xenophobia^7^, prejudice towards cultural foreignness^8^, and preference for similar others (i.e. homophily; Ref.^9^). Ambiguity around social roles and language barriers can also harm cultural outsiders’ adjustment to a new community^10^. They additionally may struggle socially if they lack experience with cultural diversity^11,12^.

Master’s of Business Administration (MBA) students represent a unique population that lend themselves to questions of how cultural outsiders fare when they join new communities. In the first year of an MBA program, students go from knowing few or none of their peers to having both deep and wide friendships, partly because a major goal of MBA programs is network-building^13^. However, as is often the case with cultural outsiders, international MBA students tend to befriend fellow international students and are thus less integrated into the wider student community^9^. The present work examines a network of MBA students that is fairly culturally diverse and enclosed. Using this sample, we consider another reason outsiders may struggle to connect: different cultural standards of social and emotional competence.

### Social connection depends on culturally relative emotional and social competencies

Being seen by others as socially and emotionally competent brings desirable outcomes, both personally and professionally. High emotional competence–being able to recognize, communicate, and regulate emotions in culturally appropriate ways–is associated with increased social support, healthier and less risky behaviors, better mental health, and career success^14,15^. Likewise, high social competence–being able to relate to and influence others, resolve conflict, and contribute to a group^15^—is associated with better financial outcomes^16^, reduced work-related emotional burnout^17^, and life satisfaction and career success^18,19^.

Part of being perceived as socially competent involves being aware of, and sufficiently adhering to, social norms. Norms regulating social interactions and emotional expression vary by nation and culture^20,21^. Cultural differences in these norms, such as when smiling is appropriate, for example, can disrupt cross-cultural interactions^22^. Avoiding eye contact^23^, openly speaking one’s mind^24^, and interrupting^25^ are markers of social competence in one culture and incompetence in another. People (cultural outsider or not) can misjudge themselves compared to others’ evaluations^26^—new cultural outsiders, who are unaware of local norms, may misunderstand how locals perceive them^10,27^. The experience of not knowing local social expectations, combined with the other challenges of being in a new environment, can threaten cultural outsiders, cause them to socially withdraw^3^, and negatively impact how they are perceived by others^8^.

### The present study

We propose that whereas cultural outsiders evaluate their social competence according to their home culture’s standards, others evaluate their social competence according to local standards, manifesting as a larger gap in self- and peer-rated competencies compared to host nationals. In the present work, we ask whether these gaps matter for social connection. If locals evaluate cultural newcomers as less socially and emotionally competent (according to local standards), this should affect their ability to become well-connected. Further, if this self-other disagreement is due to culturally relative social standards, then it should be greater for students from more culturally distant nations^28^.

The current study tested these predictions using 8 first-year cohorts in a U.S. MBA program. The sample is culturally diverse, with 35% international students. Students rated themselves and several collaborative study group peers (See “Materials and methods”) using the Emotional and Social Competencies Inventory (ESCI), which measures competence in self-management, relationship management, self-awareness, and social awareness^29^. We calculated the differences between how students rated themselves and how their peers rated them on all ESCI subscales. Because standards for social competence are culturally relative^21^, we consider the peer-ratings to be a students’ “ground truth” competence by local standards. The more a student disagrees with their peers’ judgments of their own competence, the less accurate they are in their culturally-relative self-knowledge. In secondary analyses, we used the cultural distance of international students’ home nations from the U.S.^28^ to examine the social benefits of having a cultural background more similar to the U.S.

To probe the social consequences of being judged as competent by peers, we analyzed students’ friendship networks. We quantified each student’s social embeddedness in the MBA community with network centrality^29,30^. Central individuals have a high quantity of social ties, act as critical social links^30^, are connected to well-connected or influential others, maintain long-term benefits in life and professional satisfaction^32^, can quickly share knowledge^31^, and are highly successful in job searches^32^. Centrality, like other network characteristics, is a contextualized outcome of individual-level traits (i.e., social competence, empathy^33^) and environmental factors (i.e. race^34^, network type^33,35^). This makes centrality pertinent across a variety of social contexts; it is a particularly relevant social connection metric in the present sample, as networking is a primary goal in MBA programs. Here we use network centrality as a proxy for social connectedness and test if it is predicted by nationality and emotional and social competence.

## Results

We conducted linear regression analyses in R^36^, controlling for gender and cohort year in all regression models^37^. For our mediation analyses, we utilized the sem() function from the lavaan R package^38^ and report Cliff’s delta effect sizes proportional to the total effect using the calEffSizes() function in the metaSEM R package^39^. As a complementary approach to using person-level network centrality as an outcome variable, we also report a logistic regression in which our outcome variable is the presence (or absence) of a tie between all possible student pairs in a cohort. For parallel analyses using alternate measures of social network connectedness—including social brokerage, total mutual friends, and friendship diversity—see the OSF page.

### Cultural outsiders have greater self-other disagreement in social competence

**Figure 1:**
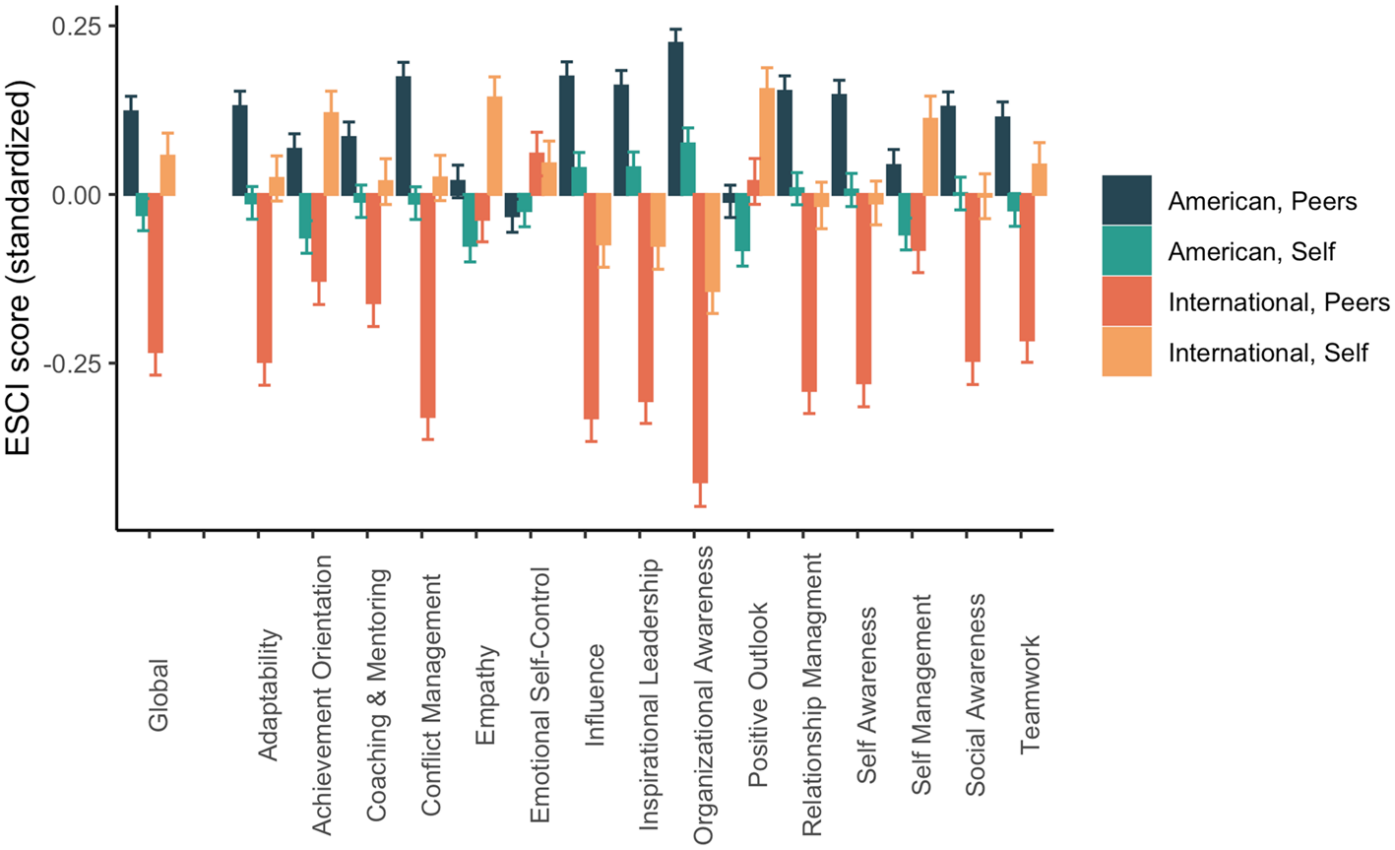
Average self- and peer-reported emotional and social competencies for international and U.S. students. Global ESCI scores are on the left, followed by scores for the 12 ESCI subscales. Self- and peer-ratings were standardized separately.

We first asked whether the discrepancy between self-reported ESCI and peer-reported ESCI was larger for international students. We regressed ESCI self-other absolute differences on nationality (contrast-coded as American students = − 0.5, international students = 0.5), controlling for gender (contrast-coded as male = − 0.5, female = 0.5) and cohort year. As hypothesized, the global self-other differences were significantly greater for international students compared to American students (Fig. 1; *b* = 2.762, *SE* = 0.895, *t*(1324) = 3.085, *p* = 0.002, *CI* = [1.005, 4.518]) when controlling for gender and cohort year. The model predicts an ESCI self-other difference of 51.18 for international students and 49.046 for American students. This pattern held significantly for 9/12 of the specific ESCI competencies (Fig. 1 and OSF page). We saw no relationship between gender or cohort year and self-other absolute differences. This difference score analysis indicates that international students do not agree with their peers regarding social and emotional competence. However, these difference scores are imperfect, since the collaborative peer groups were not all the same size (some students had more peer-ratings than others), so subsequent analyses use students’ global ESCI self- and peer-reported averages.

### Cultural outsiders are evaluated by peers as less socially competent

To clarify whether the larger self-other discrepancy for international students was driven by self- or peer-ratings, we regressed the global self-reported ESCI scores (rather than self-other absolute differences) on nationality, controlling for gender. We then did the same with peer-reported ESCI scores.

International student status neither systematically predicted the average *self*-scores on the ESCI globally nor the average self-scores for nine of the twelve competencies (See OSF). That is, international students and domestic students gave themselves similar evaluations. However, peers rated international students’ social competence (M = 3.84, SD = 0.39, min = 2.4, max = 4.82) lower than U.S. students (M = 3.97, SD = 0.35, min = 2.44, max = 4.89) globally across the ESCI (*b* = − 0.133, *SE* = 0.021, *t*(1324) = − 6.268, *p* < 0.001, *CI* = [− 0.174, − 0.091]), when controlling for gender and cohort year, neither of which predicted ESCI peer-ratings. This model predicts international students to have a global emotional and social competence of 3.83, and American students to be rated 3.976. This effect, in which international students had lower peer-ratings than American students, held across all individual competencies but three: emotional self-control, positive outlook, and empathy (Fig. 1). These three competencies are notably more intrapersonal, rather than interpersonal, in nature.

### People from more culturally distant nations have more self-other disagreement

We have established that international students were perceived as less socially and emotionally competent than their American counterparts. If these effects are driven by cultural mismatches, then we propose that international students from nations that are more culturally different from the U.S. should be perceived as lower in social competence than international students from culturally similar nations. We first regressed ESCI self-other absolute differences on international students’ cultural distance from the U.S, controlling for gender and cohort year. As hypothesized, cultural distance positively predicted larger global self-other differences (*b* = 57.565, *SE* = 20.628, *t*(427) = 2.791, *p* = 0.006, *CI* = [17.02, 98.109]), while gender and cohort year were insignificant. International students from nations more culturally distant from the United States tended to disagree more with their peers’ judgments of them, no matter their gender identities.

We then unpacked the self-other differences in the international student subsample, as we did with the full sample, by regressing self- and peer-reported global ESCI scores on cultural distance, controlling for gender, in two regressions. Cultural distance negatively predicted peer-ratings (*b* = − 1.728, *SE* = 0.459, *t*(427) = − 3.764, *p* < 0.001, *CI* = [− 2.63, − 0.826]), meaning that international students with greater cultural distance from the United States were judged as less competent by their peers than international students from more culturally similar home nations. This model predicts that international students hailing from nations with greater cultural distance from the U.S. (e.g. Armenia) would have a global ESCI peer-rating of 3.821, while international students from nations with less cultural distance from the U.S. would have a rating of 4.051. Gender and cohort year were insignificant in predicting peer-ratings. Unexpectedly, cultural distance *positively* predicted global ESCI self-ratings (*b* = 1.536, SE = 0.523, *t*(427) = 2.932, *p* = 0.004, *CI* = [0.506, 2.565]): those from nations more culturally distant from the United States tended to rate their own social and emotional competence more highly. This model predicts that international students from nations with greater cultural distance from the U.S. would have a global ESCI self-rating of 3.951, while international students from nations with less cultural distance from the U.S. would have a rating of 3.748.

### Emotional and social competence predicts social network centrality

Do self- and peer-reported emotional and social competencies predict social network position? And are these relationships moderated by being an international student? To answer these questions, we regressed social network centrality on mean-centered nationality, mean-centered self-reported global ESCI scores, and mean-centered peer-reported global ESCI scores, and their three-way interaction and all two-way interactions, controlling for gender and cohort year. At average ESCI levels, nationality negatively predicted centrality values (*b* = − 0.619 *SE* = 0.105, *t*(1318) = − 5.884, *p* < 0.001, *CI* = [− 0.083, − 0.413]), meaning that compared to their American peers, international students were less well-connected in their social networks. This model predicts that at similarly high self- and low peer-rated ESCI values, international students would have a calculated centrality of − 0.658, while American students would have a centrality value of 0.94 (centrality min. = − 4.47, max. = 6.22). Both self-reported ESCI scores (*b* = 0.426, *SE* = 0.122, *t*(1318) = 3.488, *p* =  < 0.001, *CI* = [0.184, 0.666]) and peer-reported ESCI evaluations (*b* = 0.373, SE = 0.138, *t*(1318) = 2.711, *p* = 0.007, *CI* = [0.103, 0.643]) positively predicted centrality; individuals who were rated highly competent, whether by themselves or their peers, tended to be more central to the networks. Those identifying as female tended to have greater centrality, compared to male-identifying students (*b* = 0.329, *SE* = 0.101, *t*(1318) = 3.249, *p* = 0.001, *CI* = [0.13, 0.527]). Lastly, we found a cohort effect, such that MBA students tended to have higher network centrality measures over time (*b* = 0.07, *SE* = 0.034, *t*(1318) = 2.039, *p* = 0.042, *CI* = [0.002, 0.138]).

All interaction terms were not statistically significant, suggesting the relationship between self- and peer-reported ESCI and social centrality was not meaningfully different for Americans compared to international students and the effects of self- and peer-reported competence on centrality did not moderate each other (See OSF page). Given that each peer group rated different people, there may have been variance in the scoring between all the peer groups. However, our findings were not impacted by clustering through the peer groups. For the multi-level model outputs, with a random effect specified for peer group, see Alternate Analyses in the Supplementary Materials.

### Nationality and emotional and social competence predict tie formation

The network outcome variable used in the above analyses is a principal component consisting of network centrality variables, such as betweenness centrality, eigenvector centrality, and indegree and outdegree centrality. This composite centrality variable gives us a sense of how nationality and social and emotional competence influenced students’ positions in the wider community.

But we can also ask a simpler question: do U.S. students report more friends, and are they similarly likelier to have others report them as friends? And does social and emotional competence predict the likelihood of social ties? To answer these questions, we used logistic regression to estimate whether ego or alter attributes influence the presence of a social tie among the 354,033 possible within-cohort ties. With a directed network, a person (called an *ego* in network analysis language) can be connected to an *alter* with both an outgoing tie (“they are my friend”) and an incoming tie (“they consider me a friend”). This allows us to separate the effects of predictors on the likelihood of incoming and outgoing ties separately.

We specified a multi-level logistic regression model using the glmer() function from the lme4 R library^40^ predicting the presence of ties (0 = tie absence, 1 = tie presence) with the following variables for both egos and alters separately: nationality, self-, and peer-rated ESCI scores, with all two-way and three-way interactions between them. We also included random intercepts for ego and alter. The *ego* versions of the predictor variables tell us whether the ego’s own attributes (nationality and ESCI) predict the likelihood that they report other people as their friends. The *alter* versions of the predictor variables tell us whether the alters’ attributes predict the likelihood that the ego reports them as a friend.

Ego nationality negatively predicted tie formation, meaning international students tended to nominate fewer friends than their American counterparts (*b* = − 0.191, *SE* = 0.059, *z* = − 3.26, *p* = 0.001). Ego self-reported emotional and social competence positively predicted tie formation (*b* = 0.302, *SE* = 0.069, *z* = 4.402, *p* < 0.001), meaning that those confident in their interpersonal abilities tended to nominate friends more often. Alter nationality also negatively predicted tie presence (*b* = − 0.224, *SE* = 0.033, *z* = − 6.756, *p* < 0.001); in other words, fewer people nominated international peers as friends than they did American peers. Alter peer-rated emotional and social competence positively predicted tie formation (*b* = 0.178, *SE* = 0.044, *z* = 4.011, *p* < 0.001), meaning the students considered by peers to be socially competent also had more peers reporting them as friends. All other main effects and interactions were insignificant in this model.

Although this analysis accounted for the within-person dependencies in the dyadic data with random effects, we were unable to consider the dyadic dependencies in our data from the possible reciprocal friendships. To solve this, we ran a multi-way clustering model^41^ with the clus_nway function in Stata^42^, with a parameter that would account for reciprocity, which yielded the same conclusions as the multi-level model (see OSF materials).

In sum, when the unit of analysis was social ties rather than individual participants, we found that international students reported fewer friends and were less likely to be other people’s friends compared to American students. Further, self-rated emotional and social competence predicted number of self-reported friendships, while peer-rated social competence predicted number of other-reported friendships.

### Peer-rated social and emotional competence partially mediates the relationship between nationality and centrality

We have seen that international students are perceived as relatively less emotionally and socially competent and are lower in centrality than American students. We therefore asked whether our data are consistent with a model in which peer- and self-reported ESCI scores mediate the effect of nationality on centrality. In other words, might international students achieve less prominent social network positions because they are judged as less socially and emotionally competent?

**Figure 2:**
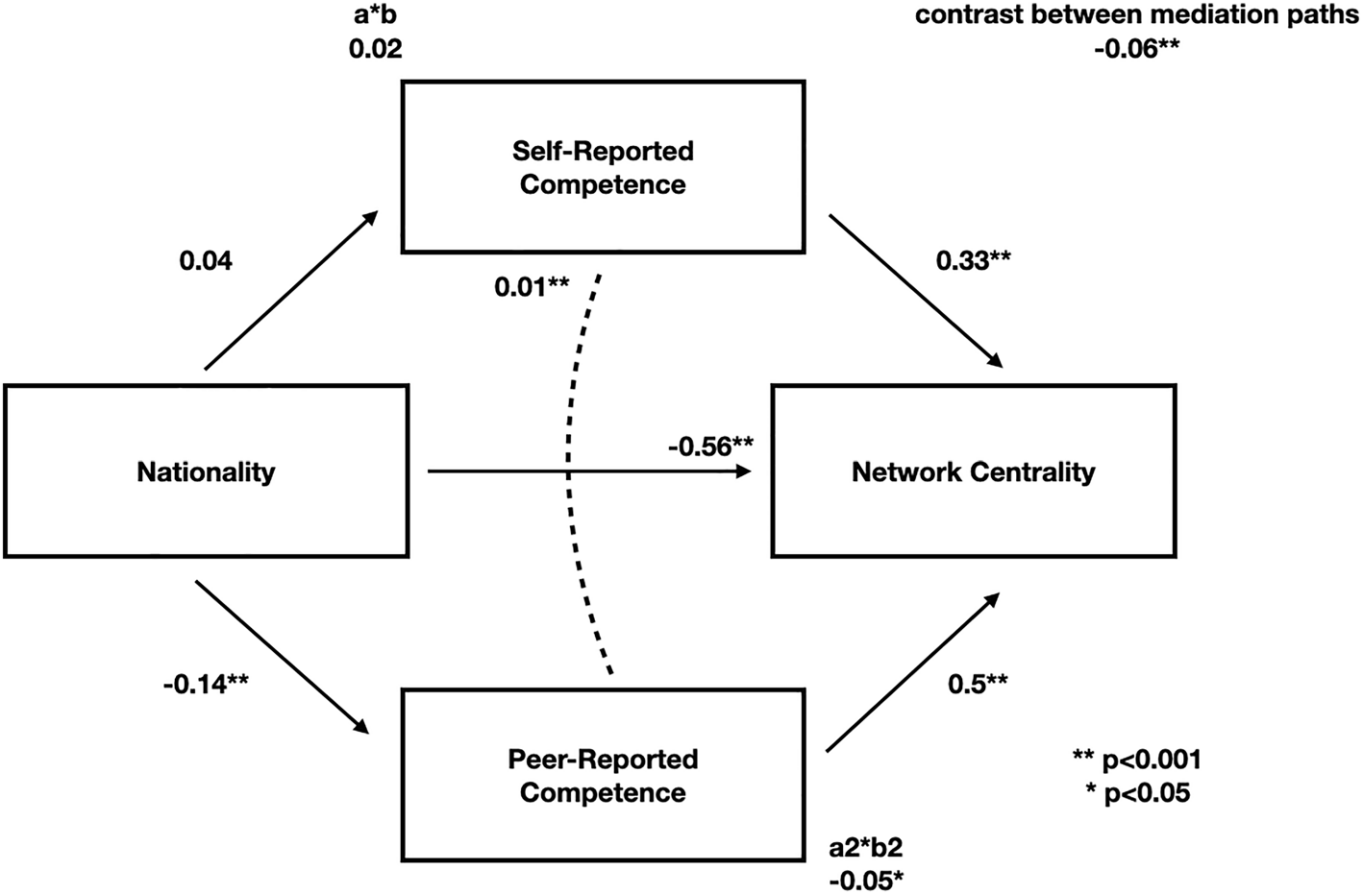
Peer-rated ESCI scores partially mediate the relationship between nationality and centrality (a2*b2 path). Indirect effect estimates are illustrated via a*b and a2*b2. Statistical significance indicated by asterisks.

We specified a structural equation model with (a) a direct path from nationality to centrality, (b) indirect paths from nationality to centrality via both self- and peer-reported global ESCI scores, and (c) covariance between self- and peer-reported ESCI scores (Fig. 2). We also estimated the statistical significance of (d) the two indirect effects, (e) the total effect (direct and indirect effects combined) and (f) the contrast between the two indirect effects.

The effect of nationality on centrality was partially mediated by ESCI peer-ratings (*b* = − 0.045, *SE* = 0.019, *p* = 0.019, *CI* = [− 0.083, − 0.007], *δ* = 0.043), but not self-ratings (Fig. 2). These indirect effects are operating in significantly different directions, as indicated by (f), *b* = − 0.06, *SE* = 0.022, *p* = 0.005, *CI* = [− 0.103, − 0.018], *δ* = − 1.281. Thus, the effect of being an international student on network centrality is partly mediated by peers’ judgments of social competence (see OSF for all model estimates).

### People from more culturally distant nations have lower centrality

**Figure 3:**
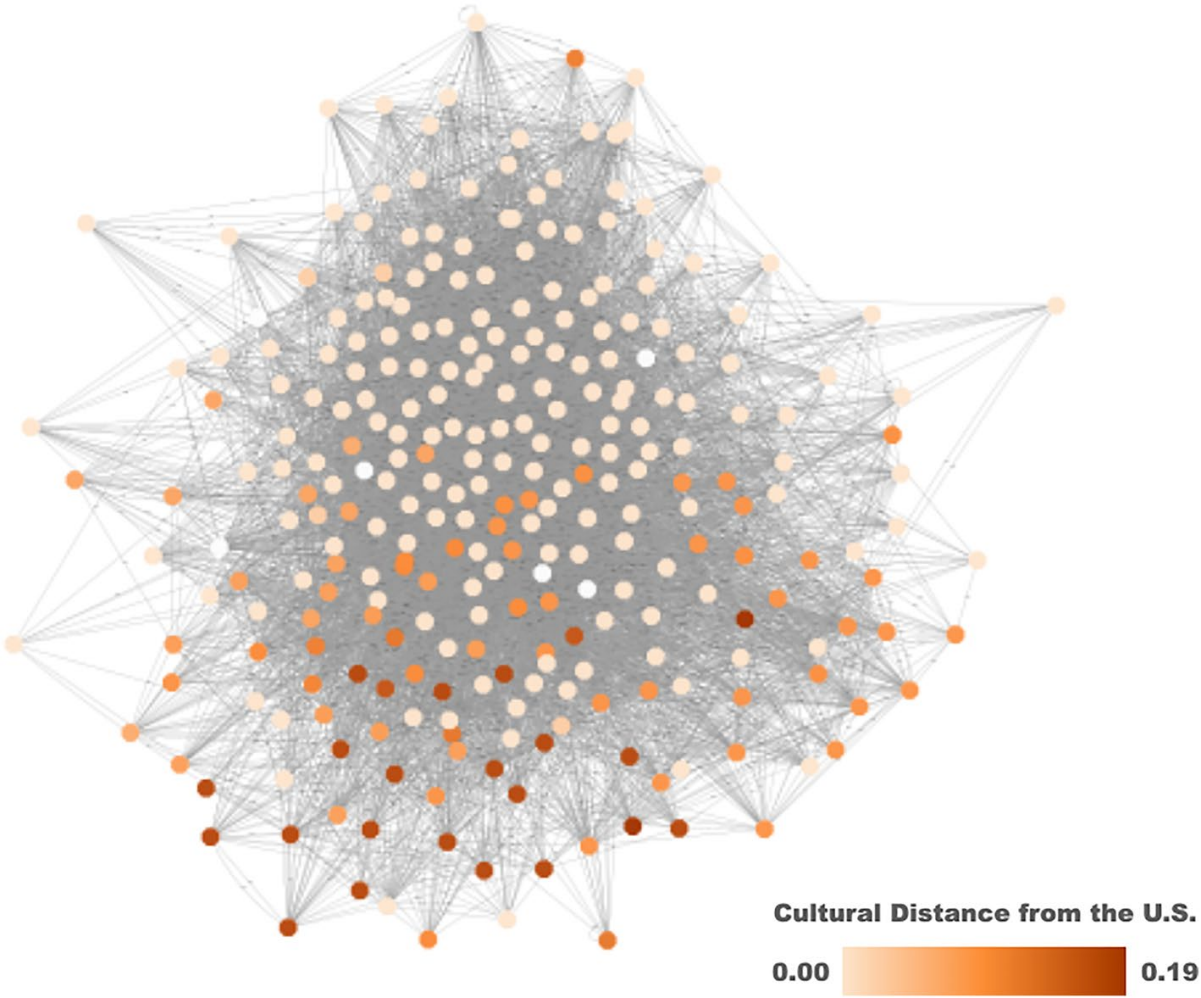
One of the eight cohort social networks analyzed, with multi-colored nodes representing students and the grey edges representing friendship ties between them. The network was plotted with a compound spring embedder layout, which serves as a proxy for social centrality. Nodes are colored according to students’ home nations’ cultural distance from the U.S.

**Figure 4:**
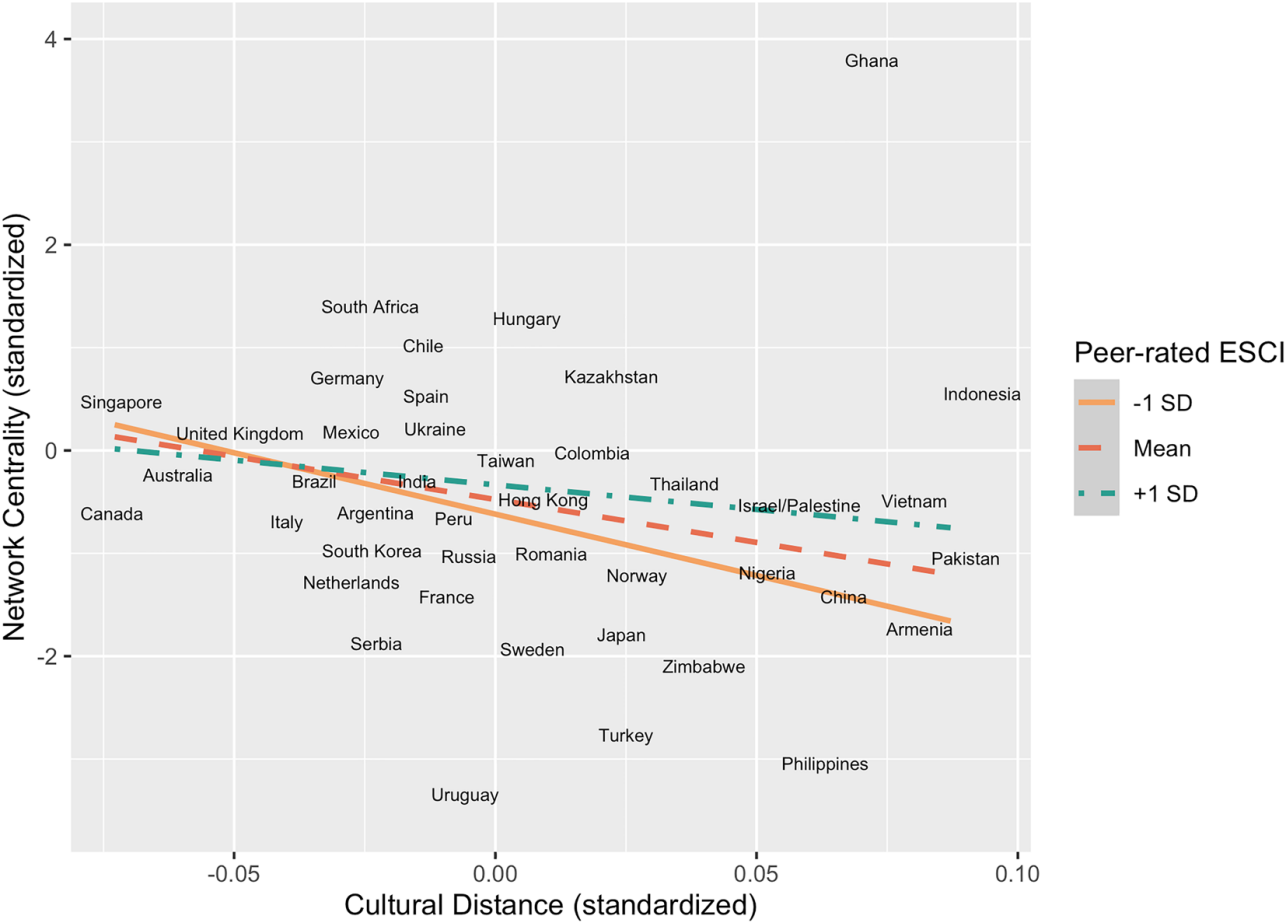
Model estimates for the two-way interaction between international students’ peer-rated ESCI scores and the cultural distance of their home nations from the U.S. predicting network centrality.

Next, we plotted the cohort networks to begin exploring whether cultural distance impacts international students’ ability to form wide-reaching social connections (for an example, see Fig. 3). We then regressed centrality on cultural distance, also including mean-centered self- and peer-rated ESCI scores, their three-way interaction with mean-centered cultural distance, and all two-way interactions, controlling for gender and cohort year. International students from more culturally distant nations tended to experience lower social centrality (*b* = − 8.347, *SE* = 1.901, *t*(421) = − 4.39,* p* < 0.001, *CI* = [− 12.085, − 4.61]). Crucially, although peer-rated ESCI did not predict centrality by itself (for a student average on self-rated ESCI scores and cultural distance), peer-ratings interacted with cultural distance, *b* = 9.08, *SE* = 4.451, *t*(421) = 2.04, *p* = 0.04, *CI* = [0.33, 17.83]. This model predicts that students from nations very culturally distant from the U.S. with high ESCI self- and peer-ratings would have a centrality score of − 0.223, whereas if they had high self-ratings and low peer-ratings, they would have a predicted centrality score of − 0.653. For international students from nations more culturally distant from the United States, having higher peer-ratings has a greater impact on network centrality (Fig. 4). The three-way and other two-way interactions, as well as the other main effects, were not significant in this model (See OSF). We replicate these findings in a multi-level model with a random effect specified for peer group (for outputs see Alternate Analyses in the Supplementary Materials).

Finally, we tested whether the international students’ data was consistent with a model in which ESCI scores mediate the effect of cultural distance on network centrality. The model structure is identical to the earlier mediation model, except the predictor is cultural distance rather than nationality. We found no evidence for self- or peer-reported ESCI mediating the relationship between cultural distance and centrality (See OSF).

These results suggest that the social impact of cultural misunderstandings is not uniform for all international students. Students from nations more culturally like the United States tended to be perceived as more socially and emotionally competent and were better-connected in their peer network. However, international students from culturally distant nations can overcome this cultural mismatch and become socially influential *if* they are perceived as socially and emotionally competent by their peers.

## Discussion

The present work suggests that cultural outsiders are less well-connected to their peers than cultural insiders, and this may be because of intergroup bias, because they behave according to different cultural definitions of social and emotional competence, or a combination of both. They misjudge their own competence (if we define “ground truth” as local consensus), are judged as less competent by their peers, and occupy less optimal social network positions compared to locals in their U.S. MBA context. Peer judgments of competence mediated the effect of being a cultural outsider on social network centrality, highlighting the network consequences of being judged as socially competent by local standards. We also found that international students’ social outcomes were impacted by their home nations’ cultural distance from the United States^28^. International students from more culturally distant nations were judged as less competent, compared to international students from nations more culturally similar to the U.S. However, international students from even culturally distant nations were more central to the network if they were judged as socially competent by their peers. A secondary analysis using multi-level logistic regression suggested that, at the local tie formation level, self-rated social and emotional competence predict *outgoing* but not *incoming* ties, but the opposite held for peer-ratings. Nationality predicted both incoming and outgoing ties, such that international students were less likely than U.S. students to report friends and be reported by peers as friends.

This study is among the first to explore self-peer disagreement throughout large social networks with such rich and ecologically valid data. Furthermore, we have used a novel measure of cultural distance^28^ to quantify social differences within our sample of cultural outsiders as they navigate their new community.

It is possible that peer judges are biased against cultural outsiders, judging them as less competent regardless of their actual behavior^8^. After all, previous research has shown that people are more accurate in recognizing and evaluating ingroup members’ anxiety^43^ and emotions^44^ compared to outgroup members’. However, anxiety and emotions have a measurable “ground truth” to compare peer judgments against: someone either has raised physiological markers or is intending to communicate an emotion, or they are not. Our measures of emotional and social competence, on the other hand, are defined by others’ evaluations and local consensus as “ground-truth”: one is not socially competent in a context if others do not believe so.

We expected a global bias against international students across all ESCI subscales, as research on intergroup perception consistently finds an outgroup homogeneity effect^45^. However, peers did not rate international students lower than U.S. students on emotional self-control, positive outlook, and empathy, which are more intrapersonal compared to other measured competencies (Fig. 1). We might interpret this as evidence that peers’ judgments were either partly based on actual behavioral observation, since intrapersonal measures are less visible to others, or outgroup-based bias. It is true that intergroup bias is not global or necessarily subject to homogeneity effects but is instead strategic across certain domains. Some work indicates that intergroup perception can be subject to strategic judgments^46,47^, although this research typically examines minimal groups unaffiliated with meaningful social identity, unlike the current sample. Further, we found cultural distance reliably predicted differences among international students: we saw that international students’ judgments of their own competence lacked agreement with their peers’ judgments of them, a gap that increased with cultural distance from the U.S. This may be reinforced by xenophobia or homophily, a networking tendency by which people prefer similar others^48^, and evaluate them favorably^49^.

People are less likely to befriend people who “seem different.” One attitudinal dimension that leads to outgroup discrimination is perceived cultural foreignness^8^. In the present work, cultural distance predicted self-other disagreement–perhaps peers were reacting to the “foreignness” of international students. This “foreignness” could contribute to what sociology terms as cultural capital, consisting of high-status cultural signals like preferences, behaviors, and attitudes, which may be leveraged in social exclusion^50^. The greater the cultural distance from the U.S., the greater gap in cultural capital international students had to traverse—this gap drives the negative peer bias against cultural outsiders. Given the pervasiveness of intergroup mistrust and bias, we speculate that our findings indicate that this cultural mismatch in what it means to be socially competent is intertwined with peer bias against international students, but future work is needed to parse the explanations for our findings.

One limitation of this study lies in the fact that data were anonymized for privacy purposes, therefore peer-raters’ nationalities are unknown. At first glance, the anonymity of the peer raters may seem like a threat to the current study’s validity, especially since perceiver identities play a role in intergroup perception^51^—what if international students were more likely to be rated by other international students? However, the peer groups were pseudorandomized with respect to nationality, so all students were uniformly likely to be rated by U.S. and international peers. Further, if peer-ratings were not anonymous, students may not have been totally honest in their ratings. Future work could probe this cultural mismatch by examining whether cultural outsiders (especially from culturally distant nations) disagree with peers when judging the competence of *other* students. If they not only misjudge their own competence, but the competence of others, as defined by local norms, that will uncover the full extent of this cultural mismatch.

Future work should also replicate the cultural distance analyses in another host nation to ensure the mechanism is cultural distance from the host population rather than cultural distance from the U.S. per se. Previous work suggests American expatriates perceived no difference in sociocultural adjustment in cultures similar and dissimilar to the U.S, although it took more time to feel proficient with perceived cultural distance^52^. Will our effects replicate in settings with different dynamics, where outsiders have more social capital? Future work should also examine the unexpected finding that the more culturally distant an international student’s home nation, the *more socially competent* they believed themselves to be. Perhaps this is a case of self-selection: only confident people would choose to attend graduate programs in culturally unfamiliar settings. Previous research shows the tendency to self-enhance, or rate oneself more favorably than peers, manifests in dimensions most valued by one’s culture^53,54^. In accordance with Fig. 1, we see that international students evaluated themselves above the mean on competencies such as achievement orientation, teamwork, and emotional self-control while American students rated themselves greater than the mean on subscales like inspirational leadership and influence. We therefore speculate our findings may combine one’s (perceived) ability and how much they value a competency, influenced both by cultural background and current social context, which future work should disentangle. Beyond varying the cultural context, future work might replicate our analyses with socioeconomic status—previous work shows that social connectedness between people of varying incomes may facilitate economic mobility^55^. Finally, we must note it is possible MBA students differ from the general population in their motivation to network and connect with others, the level at which they value emotional and social competence, or how culturally tolerant they are of social differences. Future work should test if our findings hold in other social settings.

What can (or should) communities and institutions do about cultural outsiders’ relative definitions of social and emotional competence? If peers’ negative evaluations and cultural outsiders’ inaccurate self-assessment (in their novel context) impede sociocultural integration, an intervention using peer feedback could improve social outcomes, which was the pedagogical purpose of the peer reports in the current sample. Cross-cultural training may increase intercultural competence; however, effectiveness is difficult to assess and importantly, outcomes can vary based on expatriates’ cultural distance^56^. Yet marginalization was not inevitable for the international students in the current sample, even for those more culturally distant; if they were perceived as competent by their peers, they were also well-connected.

But we argue the burden should not be placed solely on cultural outsiders, who are already adjusting to a novel environment. Learning culturally-specific norms and values takes time–and meanwhile, their peers will be establishing their advantageous positions in the network. Members of the host community should learn that interpersonal competence standards are culturally relative. Future work should explore ways to do this, as current cross-cultural awareness interventions are ineffective in creating long-term attitude change^57^. Increasing the value host nationals place on cultural diversity is one solution^58^. Structured intergroup contact is another way to improve the social connections of international students^59^. Students in our sample were assigned to intentionally diverse peer-groups for their coursework, which might eventually help them overcome cultural differences throughout their MBA program enrollment.

Finally, we do not imply that social success hinge entirely on adhering to culturally relative social standards. Perceived warmth, attractiveness, experience with cultural diversity, and personality also predict advantageous network positions^12,32,58^. Our work, however, does suggest that alignment in local cultural definitions of emotional and social competence may be important for attaining social success in the studied context.

In sum, we suggest cultural outsiders can be socially successful in a new context if they adjust to local standards of social competence or if locals broaden their definitions of social competence, perhaps through interventions meant to promote cross-cultural friendships^60,61^. Continuous opportunities for meaningful connections might help host community members avoid making dispositional attributions based on culturally relative norms.

## Method and materials

### Participants and procedure

The participants were students from 8 first year MBA cohorts from 2012 to 2019 at a private university in the United States (N = 1328). The sample size was determined by cohort size, rather than power analyses. All participants completed the ESCI^29^ for themselves and 2–7 peers in a closely collaborative study group, which were pseudorandomized regarding nationality^62^. Our sample had 244 groups in total, which ranged in size from 3 to 8 students (M = 5.45, SD = 0.72). Students met in their groups at least five times per week, oftentimes for several hours per day. We believe they were able to create meaningful impressions of their peers’ social and emotional abilities at the point of data collection. The peer-raters’ identities were anonymous to ensure validity in their evaluations. Participants also filled out social network surveys twice in their first year of the program (September, then sometime between November–April); we use data from the first timepoint, aligning with the timing of the ESCI measurement.

We included students’ data if they had self-reported ESCI values, at least one peer-reported set of ESCI values, and social networking values, excluding 300 participants from the original sample of 1628. Of the included 1328 MBA students, 869 reported their nationality as the United States and 459 reported another nationality, meaning our sample contained 35% international students from 57 countries (See Supplementary Materials Table S3). 512 identified as female, and 816 identified as male (61% male). Of those asked for their ethnicities, 671 were White non-Hispanic, 173 were Asian American Pacific Islander, 53 were Black non-Hispanic, 51 were Hispanic or Latino, 24 were multi-racial, 8 were Native American, 284 did not report, and the remaining had no response (64% White, of those reported).

All participants provided informed consent to participate in this research as part of their coursework in organizational behavior. This sample of MBA students is of interest due to the inclination to network with peers. All data collection procedures and experimental protocols ethically followed standards from the Dartmouth Committee for the Protection of Human Subjects, which serves as the Institutional Review Board and followed the relevant guidelines from the Declaration of Helsinki.

### Measures

#### Social network survey

All students were emailed a social network survey during their first year in the program, once in September (five weeks into their first term) and again later in the school year, between November and May. The first cohort completed the survey only once in November. Students indicated their social ties by checking the box next to peers’ names they considered friends (See Supplementary Materials) amid the full list of their cohort, from which we could extrapolate mutual friendships (M = 14.35, SD = 10.27, min = 0, max = 60).

Network centrality was calculated from a principal components analysis with several correlated social network measures (e.g. indegree, PageRank, and betweenness centralities; for details see Supplementary Materials Tables S1 and S2). Individuals high on centrality have a greater number of friends that they name, more people who name them as friends, and connections with other well-connected people^30^.

#### Emotional and Social Competencies Inventory

Students completed the Emotional and Social Competencies Inventory (ESCI) for themselves and anonymously for 2–7 peers from closely collaborative study groups^62^. The 70-item ESCI measures interpersonal abilities (e.g. teamwork or self-management) based on behavioral frequency on a Likert scale (1–5; Never, Rarely, Sometimes, Often, Consistently^29^). Emotional and social competencies are evaluated as observable frequencies of specific behaviors, predict real-world outcomes, reliably measured by other-assessment, and was created with American norms in mind^63^. For example items per subscale, see Supplementary Materials.

In our analyses, we examined ESCI self-other absolute differences (M = 50.62, SD = 15.49, min = 15.6, max = 146.58), indicating the size of students’ self-other disagreement. To calculate these values, we computed the following across all 70 items:

Global self-other absolute differences = ${\sum }_{n=1}^{70}|{s}_{n}-{p}_{n}|$

s = self-rating per ESCI itemp = mean of peer-ratings per ESCI item

We additionally calculated the averages of the ESCI self-reported (M = 3.91, SD = 0.43, min = 1.86, max = 5) and peer-reported (M = 3.92, SD = 0.37, min = 2.4, max = 4.89) competence scores separately.

### Cultural distance

To quantify international students’ home nations’ cultural difference from the U.S (in terms of values, beliefs, and norms), we used a recently-developed index of cultural distance^28^. The authors adapted a measure of genetic distance between populations to quantify total cultural distance across values, beliefs, and norms without assuming homogeneity within nations. Cultural distance values were computed using nationally representative and culturally transmissible data (World Values Survey) from 170,247 participants originating from 85 nations from 2005 to 2014.

We used cultural distance from the U.S. as a predictor since our sample’s MBA program is located there. International students from the nations included in calculations from Ref.^28^ were assigned their nation’s cultural distance value (0.025–0.192) to be used in analysis (See Supplementary Materials Table S3). We excluded 23 international students from analysis since their homes did not have a calculated cultural distance value.

## Supplementary Information

Supplementary Information.

## Data Availability

Complete data processing code, complete analysis code, and partial data are available on OSF (https://osf.io/a5ctb/?view_only=e7de41e9ceba40bfab073ac197efc4d3), but none of the analyses were preregistered. We do not have permission to share participant-level data publicly.

## References

[1] United Nations. International Migrant Stock | Population Division. https://www.un.org/development/desa/pd/content/international-migrant-stock (2020).

[2] Connor, P. & Budiman, A. Immigrant share in U.S. nears record high but remains below that of many other countries. *Pew Research Center* https://www.pewresearch.org/fact-tank/2019/01/30/immigrant-share-in-u-s-nears-record-high-but-remains-below-that-of-many-other-countries/ (2019).

[3] Bista, K. *Journal of International Students, 2017 Vol. 7(2)*. (OJED/STAR, 2019).

[4] Glass CR, Westmont CM (2014). Comparative effects of belongingness on the academic success and cross-cultural interactions of domestic and international students. Int. J. Intercult. Relat..

[5] Hendrickson B, Rosen D, Aune RK (2011). An analysis of friendship networks, social connectedness, homesickness, and satisfaction levels of international students. Int. J. Intercult. Relat..

[6] Ying Y-W (2002). Formation of cross-cultural relationships of Taiwanese international students in the United States. J. Community Psychol..

[7] Zamora-Kapoor A, Kovincic P, Causey C (2013). Anti-foreigner sentiment: State of the art. Sociol. Compass.

[8] Zou LX, Cheryan S (2017). Two axes of subordination: A new model of racial position. J. Pers. Soc. Psychol..

[9] Mollica KA, Gray B, Trevino LK (2003). Racial homophily and its persistence in newcomers’ social networks. Organ. Sci..

[10] Bhaskar-Shrinivas P, Harrison DA, Shaffer MA, Luk DM (2005). Input-based and time-based models of international adjustment: Meta-analytic evidence and theoretical extensions. Acad. Manage. J..

[11] Huff ST, Hanek KJ, Lee F, Brannen MY (2021). Cultural adaptation and societal context: The role of historical heterogeneity in cultural adaptation of newcomers. Int. J. Intercult. Relat..

[12] Wood A, Kleinbaum AM, Wheatley T (2022). Cultural diversity broadens social networks. J. Pers. Soc. Psychol..

[13] Baldwin TT, Bedell MD, Johnson JL (1997). The social fabric of a team-based MBA program: Network effects on student satisfaction and performance. Acad. Manage. J..

[14] Amdurer E, Boyatzis RE, Saatcioglu A, Smith ML, Taylor SN (2014). Long term impact of emotional, social and cognitive intelligence competencies and GMAT on career and life satisfaction and career success. Front. Psychol..

[15] Sarrionandia A, Mikolajczak M (2020). A meta-analysis of the possible behavioural and biological variables linking trait emotional intelligence to health. Health Psychol. Rev..

[16] Baron RA, Markman GD (2003). Beyond social capital: The role of entrepreneurs’ social competence in their financial success. J. Bus. Ventur..

[17] Carstensen B, Klusmann U (2021). Assertiveness and adaptation: Prospective teachers’ social competence development and its significance for occupational well-being. Br. J. Educ. Psychol..

[18] Emmerling R, Boyatzis R (2012). Emotional and social intelligence competencies: Cross cultural implications. Cross Cult. Manag. Int. J..

[19] Miao C, Humphrey RH, Qian S (2018). A cross-cultural meta-analysis of how leader emotional intelligence influences subordinate task performance and organizational citizenship behavior. J. World Bus..

[20] Poulou MS, Bassett HH, Denham SA (2018). Teachers’ perceptions of emotional intelligence and social-emotional learning: Students’ emotional and behavioral difficulties in U.S. and Greek preschool classrooms. J. Res. Child. Educ..

[21] Stankov L (2015). Four GLOBE dimensions of perceived social norms in 33 countries. Learn. Individ. Differ..

[22] Wiseman RL, Pan X (2004). Smiling in the People’s Republic of China and the United States: Status and situational influences on the social appropriateness of smiling. Intercult. Commun. Stud..

[23] Uono S, Hietanen JK (2015). Eye contact perception in the west and east: A cross-cultural study. PLoS ONE.

[24] Lee W, Detenber BH, Willnat L, Aday S, Graf J (2004). A cross-cultural test of the spiral of silence theory in Singapore and the United States. Asian J. Commun..

[25] Murata K (1994). Intrusive or co-operative? A cross-cultural study of interruption. J. Pragmat..

[26] Vazire S (2010). Who knows what about a person? The self–other knowledge asymmetry (SOKA) model. J. Pers. Soc. Psychol..

[27] Eng, L. A. Viewing ourselves and others: differences, disconnects and divides among locals and immigrants in Singapore. 44 (2012).

[28] Muthukrishna M (2020). Beyond western, educated, industrial, rich, and democratic (WEIRD) psychology: Measuring and mapping scales of cultural and psychological distance. Psychol. Sci..

[29] Fang R (2015). Integrating personality and social networks: A meta-analysis of personality, network position, and work outcomes in organizations. Organ. Sci..

[30] Sasovova Z, Mehra A, Borgatti SP, Schippers MC (2010). Network churn: The effects of self-monitoring personality on brokerage dynamics. Adm. Sci. Q..

[31] Baek SI, Bae SH (2019). The effect of social network centrality on knowledge sharing. J. Serv. Sci. Res..

[32] Brass, D. J. Taking Stock of Networks and Organizations: A Multilevel Perspective. 24 (2022).

[33] Morelli SA, Ong DC, Makati R, Jackson MO, Zaki J (2017). Empathy and well-being correlate with centrality in different social networks. Proc. Natl. Acad. Sci..

[34] Kim HK, McKenry PC (1998). Social networks and support: A comparison of African Americans, Asian Americans, Caucasians, and Hispanics. J. Comp. Fam. Stud..

[35] Inkpen AC, Tsang EW (2005). Social capital, networks, and knowledge transfer. Acad. Manag. Rev..

[36] Team, R. C. R: A Language and Environment for Statistical Computing http://www.R-Proj.Org (2014).

[37] Taylor SN, Hood JN (2011). It may not be what you think: Gender differences in predicting emotional and social competence. Hum. Relat..

[38] Rosseel Y (2017). Package ‘lavaan’. Retr. June.

[39] Cheung MW-L (2015). metaSEM: An R package for meta-analysis using structural equation modeling. Front. Psychol..

[40] Bates D, Mächler M, Bolker B, Walker S (2015). Fitting linear mixed-effects models using lme4. J. Stat. Softw..

[41] Cameron AC, Gelbach JB, Miller DL (2011). Robust inference with multiway clustering. J. Bus. Econ. Stat..

[42] Kleinbaum AM, Stuart TE, Tushman ML (2013). Discretion within constraint: Homophily and structure in a formal organization. Organ. Sci..

[43] Gray HM, Mendes WB, Denny-Brown C (2008). An in-group advantage in detecting intergroup anxiety. Psychol. Sci..

[44] Kang S-M, Lau AS (2013). Revisiting the out-group advantage in emotion recognition in a multicultural society: Further evidence for the in-group advantage. Emotion.

[45] Boldry JG, Gaertner L, Quinn J (2007). Measuring the measures: A meta-analytic investigation of the measures of outgroup homogeneity. Group Process. Intergroup Relat..

[46] Boldry JG, Gaertner L (2006). Separating status from power as an antecedent of intergroup perception. Group Process. Intergroup Relat..

[47] Ellemers N, Van Rijswijk W, Roefs M, Simons C (1997). Bias in intergroup perceptions: Balancing group identity with social reality. Pers. Soc. Psychol. Bull..

[48] McPherson M, Smith-Lovin L, Cook JM (2001). Birds of a feather: Homophily in social networks. Annu. Rev. Sociol..

[49] Ostroff C, Atwater LE, Feinberg BJ (2004). Understanding SELF-OTHER agreement: A look at rater and ratee characteristics, context, and outcomes. Pers. Psychol..

[50] Lamont M, Lareau A (1988). Cultural capital: Allusions, gaps and glissandos in recent theoretical developments. Sociol Theory.

[51] Rubin M, Badea C (2012). They’re all the same!... But for several different reasons: A review of the multicausal nature of perceived group variability. Curr. Dir. Psychol. Sci..

[52] Selmer J (2007). Which is easier, adjusting to a similar or to a dissimilar culture? American business expatriates in Canada and Germany. Int. J. Cross Cult. Manag..

[53] Sedikides C, Gaertner L, Vevea JL (2007). Evaluating the evidence for pancultural self-enhancement. Asian J. Soc. Psychol..

[54] Sedikides C, Gaertner L, Vevea JL (2005). Pancultural self-enhancement reloaded: A meta-analytic reply to Heine (2005). J. Personal. Soc. Psychol..

[55] Chetty R (2022). Social capital I: Measurement and associations with economic mobility. Nature.

[56] Joshua-Gojer AE (2012). Cross-cultural training and success versus failure of expatriates. Learn. Perform. Q..

[57] Hill ME, Augoustinos M (2001). Stereotype change and prejudice reduction: Short- and long-term evaluation of a cross–cultural awareness programme. J. Community Appl. Soc. Psychol..

[58] Grigoryan L, Schwartz SH (2021). Values and attitudes towards cultural diversity: Exploring alternative moderators of the value–attitude link. Group Process. Intergroup Relat..

[59] Amir Y (1969). Contact hypothesis in ethnic relations. Psychol. Bull..

[60] Allen JP, Narr RK, Nagel AG, Costello MA, Guskin K (2021). The connection project: Changing the peer environment to improve outcomes for marginalized adolescents. Dev. Psychopathol..

[61] Johnson DW, Johnson RT (2013). 1. The three Cs of reducing prejudice and discrimination. Reducing Prejudice and Discrimination.

[62] Kleinbaum AM, Jordan AH, Audia PG (2015). An altercentric perspective on the origins of brokerage in social networks: How perceived empathy moderates the self-monitoring effect. Organ. Sci..

[63] Boyatzis RE (2016). Commentary on Ackley (2016): Updates on the ESCI as the behavioral level of emotional intelligence. Consult. Psychol. J. Pract. Res..

