# Supplementary material for: Social networks are shaped by culturally contingent assessments of social competence
Source: Sci Rep. 2023 May 17;13:7974. doi: 10.1038/s41598-023-34723-6 (PMC10192412; doi:10.1038/s41598-023-34723-6)
Supplement: Supplementary file 1 — Supplementary Information. [file 41598_2023_34723_MOESM1_ESM.docx]

**Supplementary Materials for Social Networks are Shaped by Culturally Contingent Assessment of Social Competence**

**ESCI example items.** The ESCI examines 12 learned competencies, outlined with an example item each: emotional self-awareness (“Able to describe how own feelings affect own actions”), emotional self-control (“Remains calm in stressful situations”), adaptability (“Adapts to shifting priorities and rapid change”), achievement orientation (“Initiates actions to improve own performance”), positive outlook (“Views the future with hope”), empathy (“Understands another person’s motivation”), organizational awareness (“Understands the team’s or organization’s unspoken rules”), influence (“Convinces others by appealing to their self-interest”), coaching and mentoring (“Cares about others and their development”), conflict management (“Resolves conflict by de-escalating the emotions in a situation”), teamwork (“Works well in teams by encouraging cooperation”), and inspirational leadership (“Leads by bringing out the best in people”).

**Social network survey question.** This survey consisted of a cohort roster and the following prompt: “Consider the people with whom you like to spend your free time. Since you arrived at [institution name], who are the classmates you have been with most often for informal social activities, such as going out to lunch, dinner, films, visiting one another’s homes, exercising together, and so on?” (adapted from (Burt, 1992).

**Social network calculations.** We calculated the following variables for each participant (for each, we note whether we used directed or undirected graphs in our calculations): structural constraint (which we later reversed to become brokerage for the sake of clarity), indegree centrality, outdegree centrality, eigenvector centrality, betweenness centrality, pagerank, and clustering coefficient (see Table S1 for descriptions). All variables were positively skewed so we log-transformed them (log_e_(variable+1)).

| **Social Network Variable** | **Description** | **Network Centrality (PC1)** |
| --- | --- | --- |
| Constraint | Degree to which person's friends are redundant, versus connecting otherwise unconnected parts of the network (U). For the primary analyses we reversed this to become **brokerage,** but kept it in its original state for the PCA | -0.356 |
| Indegree Centrality | Number of incoming ties (D) | 0.351 |
| Outdegree Centrality | Number of outgoing ties (D) | 0.439 |
| Eigenvector Centrality | Connectedness to well-connected others (U) | 0.421 |
| Betweenness Centrality | Extent to which person acts as a bridge on the shortest path between all other pairs of people (U) | 0.416 |
| Pagerank | Similar to eigenvector but corrects for inflation of centrality scores for peripheral friends of highly-central people (U) | 0.450 |
| Clustering Coefficient | Probability that a person's friends are also friends with each other (i.e., cliquish) (U) | -0.062 |

***Table S1.*** *The 7 social network variables computed for all participants and their loadings onto the first principal component, which we named Network Centrality based on the loadings. (D) indicates variables derived from a directed network graph and (U), variables derived from an undirected graph that included only mutual ties. Color indicates the direction and strength of the variable loadings (green = positive and red = negative).*

We then wanted to explore other social network outcomes for MBA students, based on their self- and peer-rated social and emotional competence. But because some of the above social network variables are highly correlated, we reduced dimensionality using a principal component analysis of the 7 variables using the prcomp function in the stats package for R. We therefore used them as the social connectivity outcome measure in the main text (see Table S1 for variables and loadings). The first principal component, which we refer to as Network Centrality, loads onto the social network centrality measures along with constraint: people high on this component report having more friends, have more people reporting them as friends, connect well-connected others, and bridge otherwise unconnected parts of the network, acting as social brokers. These social network measures were correlated with one another (Table S2).

|  | **Constraint** | **Indegree (Log)** | **Outdegree (Log)** | **Eigenvector** | **Betweenness** | **Pagerank** | **Clustering** |
| --- | --- | --- | --- | --- | --- | --- | --- |
| **Constraint** | 1.000 | 0.570 | 0.786 | 0.577 | 0.788 | 0.633 | 0.126 |
| **Indegree (Log)** | 0.570 | 1.000 | 0.415 | 0.600 | 0.410 | 0.515 | 0.084 |
| **Outdegree (Log)** | 0.786 | 0.415 | 1.000 | 0.713 | 0.776 | 0.787 | 0.089 |
| **Eigenvector** | 0.577 | 0.600 | 0.713 | 1.000 | 0.568 | 0.820 | 0.060 |
| **Betweenness** | 0.788 | 0.410 | 0.776 | 0.568 | 1.000 | 0.814 | 0.298 |
| **Pagerank** | 0.633 | 0.515 | 0.787 | 0.820 | 0.814 | 1.000 | 0.115 |
| **Clustering** | 0.126 | 0.084 | 0.089 | 0.060 | 0.298 | 0.115 | 1.000 |

***Table S2.*** *The 7 social network variables’ correlations with one another.*

**Cultural Distance.** The following table describes the students in the present sample, by their home country, cultural distance value from the United States (Muthukrishna et al., 2020), and frequency within the sample (Table S3).

| **Nationality** | **Cultural Distance**  **(from the U.S.)** | **Frequency in N** |
| --- | --- | --- |
| No Response | NA | 8 |
| Argentina | 0.072 | 11 |
| Armenia | 0.175 | 2 |
| Australia | 0.033 | 6 |
| Austria | NA | 1 |
| Botswana | NA | 1 |
| Brazil | 0.07 | 41 |
| Canada | 0.025 | 24 |
| Chile | 0.08 | 1 |
| China | 0.17 | 68 |
| Colombia | 0.112 | 5 |
| Cote D'Ivoire | NA | 1 |
| Croatia | NA | 1 |
| Czech Republic | NA | 3 |
| Denmark | NA | 1 |
| France | 0.084 | 3 |
| Germany | 0.079 | 5 |
| Ghana | 0.174 | 1 |
| Greece | NA | 2 |
| Haiti | NA | 1 |
| Hong Kong | 0.098 | 9 |
| Hungary | 0.108 | 2 |
| India | 0.087 | 97 |
| Indonesia | 0.19 | 1 |
| Ireland | NA | 2 |
| Israel/Palestine | 0.152 | 2 |
| Italy | 0.062 | 4 |
| Jamaica | NA | 1 |
| Japan | 0.118 | 10 |
| Kazakhstan | 0.116 | 2 |
| Kenya | NA | 2 |
| Mexico | 0.075 | 23 |
| Sweden | 0.109 | 1 |
| Taiwan | 0.104 | 3 |
| Thailand | 0.139 | 1 |
| Turkey | 0.127 | 4 |
| Uganda | NA | 1 |
| Ukraine | 0.085 | 3 |
| United Kingdom | 0.056 | 10 |
| United States | 0 | 869 |
| Uruguay | 0.088 | 1 |
| Vietnam | 0.174 | 3 |
| Zimbabwe | 0.132 | 5 |

***Table S3.*** *The nations represented by the international students in the present sample, their cultural distance value from the U.S., and the number of students by nation.*

*Alternate Analyses*

*Nationality, emotional and social competence, and centrality.*

Given that each peer group rated different people, there may have been variance in the scoring between all the peer groups. We ran a multi-level model using the lme4 package in R. We regressed social network centrality on mean-centered nationality, mean-centered self- and peer-reported ESCI scores, their interactions, and a random effect for peer group, while controlling for gender. We did not control for cohort year, since we specified a random effect for groups over time. We see results identical to those reported in the main text: international students tended to have lower levels of centrality (*b* = -.617, *SE* = .105, *t*(1167) = -5.882, *p* < .001), while those identifying as female had higher levels of centrality (*b* = .351, *SE* = .1, *t*(1165) = 3.502, *p* = < .001). Both self- (*b* = .423, *SE* = .123, *t*(1317) = 3.5, *p* < .001) and peer-reported emotional and social competence (*b* = .381, *SE* = .14, *t*(930) = 2.733, *p* = .006) positively predicted centrality. Those who were considered more emotionally and socially competent, by their peers’ ratings or their own, tended to hold more central positions in their cohort networks. No interaction effects were significant in this model. The intraclass correlation for the peer groups was 0.016, suggesting that our effects are not explained by groups of peer-raters.

*Cultural distance, emotional and social competence, and centrality.*

For our subset of just international students, we regressed social network centrality on mean-centered cultural distance, mean-centered self- and peer-reported ESCI scores, their interactions, and a random effect for peer group, while controlling for gender. We did not control for cohort year, since we specified a random effect for groups over time. We see results identical to those reported in the main text: international students from nations less similar to the U.S. tended to have lower levels of centrality (*b* = -.344, *SE* = .079, *t*(403) = -4.356, *p* < .001) compared to those from nations with low cultural distance to the U.S.SDelf-reported emotional and social competence (*b* = .464, *SE* = .174, *t*(419) = 2.662, *p* = .008) positively predicted centrality. Those who rated their own social and emotional competence highly, tended to hold more central positions in their cohort networks. Lastly, we see that ESCI peer-ratings interacted with cultural distance (*b* = .37, *SE* = .185, *t*(411) = 2.003, *p* = .046), such that students from nations culturally dissimilar from the U.S. who had high peer-rated emotional and social competence were more central to their cohort networks. The other interaction effects were significant in this model. The intraclass correlation for the peer groups was 0.032, suggesting that our effects are not explained by groups of peer-raters.

**References**

Burt, R. (1992). *Structural holes: The social structure of competition (Harvard, MA, Harvard University Press)*.

Muthukrishna, M., Bell, A. V., Henrich, J., Curtin, C. M., Gedranovich, A., McInerney, J., & Thue, B. (2020). Beyond Western, Educated, Industrial, Rich, and Democratic (WEIRD) Psychology: Measuring and Mapping Scales of Cultural and Psychological Distance. *Psychological Science*, *31*(6), 678–701. https://doi.org/10.1177/0956797620916782
